# Bis(cinnamato-κ*O*)(1,10-phenanthroline-κ^2^ *N*,*N*′)copper(II)

**DOI:** 10.1107/S1600536813010350

**Published:** 2013-04-20

**Authors:** Meriem Benslimane, Yasmine Kheira Redjel, Hocine Merazig, Jean-Claude Daran

**Affiliations:** a Unité de Recherche de Chimie de l’Environnement et Moléculaire Structurale (CHEMS), Faculté des Sciences Exactes, Département de Chimie, Université de Constantine 1, 25000 Constantine, Algeria; b Laboratoire de Chimie de Coordination, UPR-CNRS 8241, 205 route de Narbonne, 31077 Toulouse Cedex 4, France

## Abstract

The title mononuclear Cu^II^ complex, [Cu(C_9_H_7_O_2_)_2_(C_12_H_8_N_2_)], is comprised of a Cu^II^ cation, two cinnamate (*L* ^−^) ligands and a 1,10-phenanthroline (phen) ligand. The Cu^II^ atom and phen ligand lie on a twofold rotation axis. The Cu^II^ atom is coordinated by two O atoms from two carboxyl­ate groups of two (*L* ^−^) ligands and two N atoms from one phen ligand, exhibiting a distorted square-planar geometry. In the crystal, mol­ecules are assembled into supra­molecular chains parallel to the *c* axis through weak C—H⋯O hydrogen bonds involving the phen and cinnamate ligands.

## Related literature

1,10-Phenanthroline is of great inter­est in the field of supra­molecular chemistry as it can form C—H⋯O or C—H⋯N hydrogen bonds and π–π stacking inter­actions (Liu *et al.*, 2004 ▶; Wang *et al.*, 2003 ▶), which can effectively result in one-dimensional or two-dimensional networks.
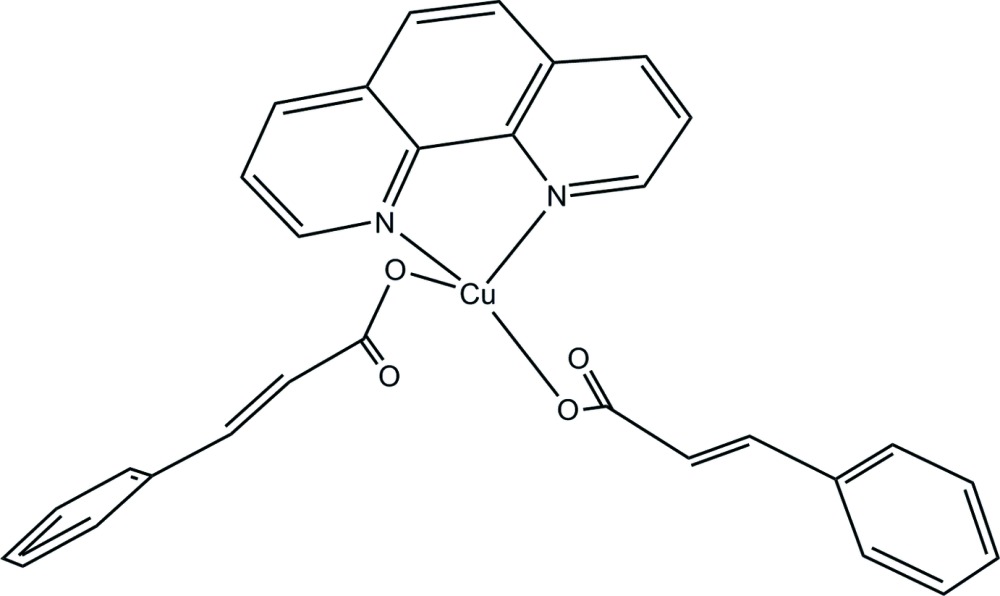


## Experimental

### 

#### Crystal data

[Cu(C_9_H_7_O_2_)_2_(C_12_H_8_N_2_)]
*M* *_r_* = 538.04
Monoclinic, 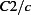

*a* = 24.486 (5) Å
*b* = 9.986 (5) Å
*c* = 10.710 (5) Å
β = 109.623 (5)°
*V* = 2466.7 (18) Å^3^
*Z* = 4
Mo *K*α radiation
μ = 0.93 mm^−1^
*T* = 180 K
0.35 × 0.17 × 0.09 mm

#### Data collection

Bruker APEXII CCD diffractometer
Absorption correction: multi-scan (*SADABS*; Sheldrick, 2008*a* ▶) *T*_min_ = 0.797, *T*_max_ = 1.000
6897 measured reflections
2172 independent reflections
1698 reflections with *I* > 2σ(*I*)
*R*_int_ = 0.050

#### Refinement

*R*[*F* ^2^ > 2σ(*F* ^2^)] = 0.039
*wR*(*F* ^2^) = 0.100
*S* = 1.08
2172 reflections
168 parameters
H-atom parameters constrained
Δρ_max_ = 0.37 e Å^−3^
Δρ_min_ = −0.37 e Å^−3^

### 

Data collection: *APEX2* (Bruker, 2012 ▶); cell refinement: *SAINT* (Bruker, 2012 ▶); data reduction: *SAINT*; program(s) used to solve structure: *SIR92* (Altomare *et al.*, 1993 ▶); program(s) used to refine structure: *SHELXL97* (Sheldrick, 2008*b* ▶); molecular graphics: *ORTEPIII* (Burnett & Johnson, 1996 ▶) and *ORTEP-3 for Windows* (Farrugia, 2012 ▶); software used to prepare material for publication: *SHELXL97*.

## Supplementary Material

Crystal structure: contains datablock(s) global, I. DOI: 10.1107/S1600536813010350/vm2193sup1.cif

Structure factors: contains datablock(s) I. DOI: 10.1107/S1600536813010350/vm2193Isup2.hkl

Additional supplementary materials:  crystallographic information; 3D view; checkCIF report

## Figures and Tables

**Table 1:** Hydrogen-bond geometry (Å, °)

*D*—H⋯*A*	*D*—H	H⋯*A*	*D*⋯*A*	*D*—H⋯*A*
C14—H14⋯O2^i^	0.93	2.49	3.176 (5)	131

Symmetry code: (i) 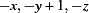.

**Fig. 1.**
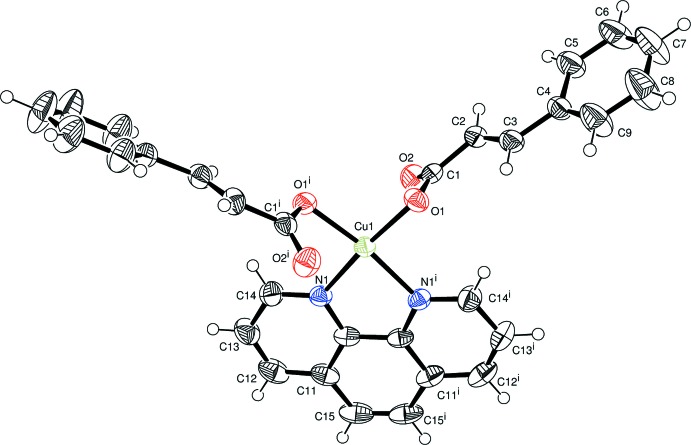
The molecular structure of the title compound, showing the atom-numbering scheme. Displacement ellipsoids are drawn at the 50% probability level and H atoms are shown as small spheres of arbitrary radii [symmetry code: (i): -x, y, -z + 1/2].

**Fig. 2.**
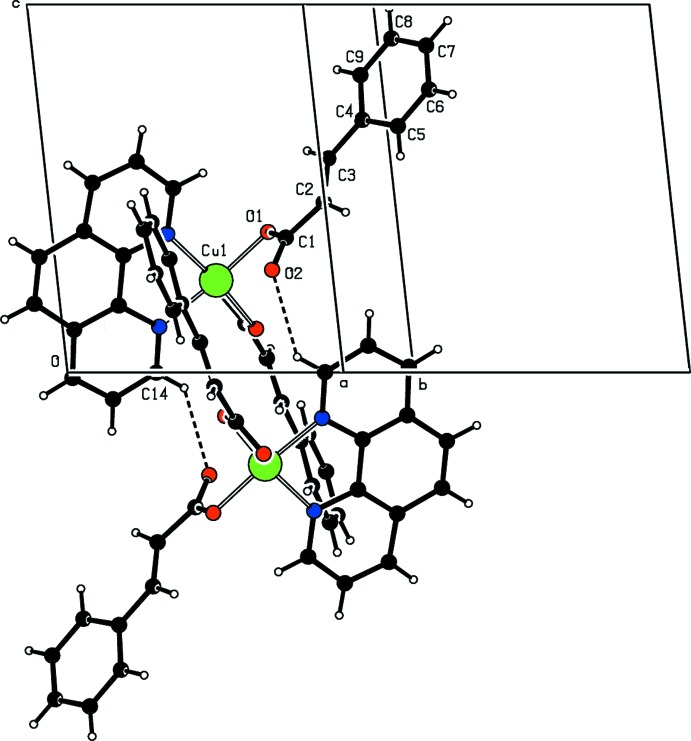
The crystal packing of the title compound, viewed down the a axis. Hydrogen bonds are shown as dashed lines.

## supplementary crystallographic information

### Comment

The mononuclear metal complexes of the chelating bidentate 1,10-phenanthroline (phen) and 2,2'-bipyridine (bipy) ligands are well known in the literature, and have been used in many fields. In the realm of coordination polymers, these complexes have been employed as coordination acceptor nodes for the construction of low dimensional polymer-based magnets exhibiting long-range magnetic ordering and spin crossover transitions. 1,10-Phenanthroline is of great interest in the field of supramolecular chemistry, because it can bring C—H···O or C—H···N hydrogen bonds and π-π stacking interactions (Liu *et al.*, 2004 and Wang *et al.*, 2003), which can effectively result in one-dimensional or two-dimensional networks. We report here the preparation and crystal structure of the title compound,[Cu(C_9_H_7_O_2_)_2_(C_12_H_8_N_2_)].

The asymmetric unit contains a half Cu^II^ cation, a half phen ligand and one cinnamic ligand (*L*^-^: C_6_H_5_—CH=CH-COO^-^). The Cu^II^ atom lies on a twofold rotation axis. In the complex, two equivalent *L*^-^ anions function as monodentate ligands, while one phen molecule functions as a terminal ligand adopting the expected chelating mode to coordinate with one Cu^II^ ion, forming a mononuclear unit. The Cu^II^ ion is coordinated by two O atoms (O1, O1^i^, symmetry code (i): -*x*, *y*, -*z* + 1/2) from two cinnamic ligands, two N atoms (N1,N1^i^) from 1,10-phenanthroline molecules, exhibiting essentially distorted square planar geometry (Fig. 1). The Cu–N/O bonds distances are 2.018 (3) Å and 1.948 (3) Å, respectively. The carboxylate group shows a distortion from the molecular plane; the dihedral angle between the mean-plane (C3—C9) and the carboxlate group (O1/C1/O2) is 25.8 (4)°. The two carboxylate groups are almost perpendicular to one another with a dihedral angle of 78.9 (6)°.

In the crystal, molecules are assembled into one dimensional supramolecular chains parallel to the *c* axis through weak C—H···O hydrogen bonds involving the phen and carboxylate ligands (Table 1, Fig. 2).

### Experimental

A methanol solution (5 ml) of phen (0.046 mg, 0.25 mmol) and cinnamic acid (0.07 mg, 0.5 mmol) were added dropwise to a methanol solution of (5 ml) CuSO_4_.5H_2_O (0.058 mg, 0.25 mmol) with constant stirring during 1 h. The mixture was then filtered and the filtrate allowed to stand for 10 days, after which small blue block-like crystals of the title complex were obtained.

### Refinement

The C-bound hydrogen atoms were placed in geometrically idealized positions and constrained to ride on their parent atom positions with a C–H distances of 0.93 Å and with *U*_iso_(H) = 1.2*Ueq*(C).

### Crystal data

**Table:**

[Cu(C_9_H_7_O_2_)_2_(C_12_H_8_N_2_)]	*F*(000) = 1108
*M**_r_* = 538.04	*D*_x_ = 1.449 Mg m^−^^3^
Monoclinic, *C*2/*c*	Mo *K*α radiation, λ = 0.71073 Å
Hall symbol: -C 2yc	Cell parameters from 1791 reflections
*a* = 24.486 (5) Å	θ = 2.8–24.9°
*b* = 9.986 (5) Å	µ = 0.93 mm^−^^1^
*c* = 10.710 (5) Å	*T* = 180 K
β = 109.623 (5)°	Platelet, blue
*V* = 2466.7 (18) Å^3^	0.35 × 0.17 × 0.09 mm
*Z* = 4	

### Data collection

**Table:**

Bruker APEXII CCD diffractometer	2172 independent reflections
Radiation source: fine-focus sealed tube	1698 reflections with *I* > 2σ(*I*)
Graphite monochromator	*R*_int_ = 0.050
φ and ω scans	θ_max_ = 25.1°, θ_min_ = 3.2°
Absorption correction: multi-scan (*SADABS*; Sheldrick, 2008*a*)	*h* = −29→28
*T*_min_ = 0.797, *T*_max_ = 1.000	*k* = −11→11
6897 measured reflections	*l* = −12→12

### Refinement

**Table:**

Refinement on *F*^2^	Primary atom site location: structure-invariant direct methods
Least-squares matrix: full	Secondary atom site location: difference Fourier map
*R*[*F*^2^ > 2σ(*F*^2^)] = 0.039	Hydrogen site location: inferred from neighbouring sites
*wR*(*F*^2^) = 0.100	H-atom parameters constrained
*S* = 1.08	*w* = 1/[σ^2^(*F*_o_^2^) + (0.0509*P*)^2^] where *P* = (*F*_o_^2^ + 2*F*_c_^2^)/3
2172 reflections	(Δ/σ)_max_ < 0.001
168 parameters	Δρ_max_ = 0.37 e Å^−^^3^
0 restraints	Δρ_min_ = −0.37 e Å^−^^3^

### Special details

**Table:**

Geometry. All s.u.'s (except the s.u. in the dihedral angle between two l.s. planes) are estimated using the full covariance matrix. The cell s.u.'s are taken into account individually in the estimation of s.u.'s in distances, angles and torsion angles; correlations between s.u.'s in cell parameters are only used when they are defined by crystal symmetry. An approximate (isotropic) treatment of cell s.u.'s is used for estimating s.u.'s involving l.s. planes.
Refinement. Refinement of *F*^2^ against ALL reflections. The weighted *R*-factor *wR* and goodness of fit *S* are based on *F*^2^, conventional *R*-factors *R* are based on *F*, with *F* set to zero for negative *F*^2^. The threshold expression of *F*^2^ > σ(*F*^2^) is used only for calculating *R*-factors(gt) *etc*. and is not relevant to the choice of reflections for refinement. *R*-factors based on *F*^2^ are statistically about twice as large as those based on *F*, and *R*- factors based on ALL data will be even larger.

### Fractional atomic coordinates and isotropic or equivalent isotropic displacement parameters (Å^2^)

**Table:**

	*x*	*y*	*z*	*U*_iso_*/*U*_eq_	
Cu1	0.0000	0.45859 (4)	0.2500	0.03280 (19)	
O1	0.04096 (9)	0.59098 (18)	0.3818 (2)	0.0401 (5)	
O2	0.10280 (9)	0.5414 (2)	0.2789 (2)	0.0519 (6)	
N1	−0.03295 (11)	0.3059 (2)	0.1237 (2)	0.0347 (6)	
C1	0.09029 (13)	0.6012 (3)	0.3663 (3)	0.0370 (7)	
C4	0.17627 (13)	0.7909 (3)	0.6862 (3)	0.0395 (7)	
C3	0.13359 (13)	0.7143 (3)	0.5815 (3)	0.0408 (7)	
H3	0.1023	0.6808	0.6023	0.049*	
C10	−0.01823 (13)	0.1841 (2)	0.1824 (3)	0.0356 (7)	
C2	0.13404 (13)	0.6868 (3)	0.4616 (3)	0.0404 (7)	
H2	0.1635	0.7232	0.4355	0.049*	
C11	−0.03787 (14)	0.0630 (3)	0.1159 (3)	0.0441 (8)	
C15	−0.01798 (15)	−0.0590 (3)	0.1870 (4)	0.0557 (10)	
H15	−0.0303	−0.1404	0.1446	0.067*	
C13	−0.08989 (16)	0.1950 (3)	−0.0730 (3)	0.0532 (9)	
H13	−0.1148	0.2016	−0.1601	0.064*	
C14	−0.06717 (14)	0.3102 (3)	−0.0004 (3)	0.0452 (8)	
H14	−0.0766	0.3932	−0.0415	0.054*	
C12	−0.07512 (16)	0.0728 (3)	−0.0149 (4)	0.0553 (9)	
H12	−0.0900	−0.0045	−0.0627	0.066*	
C5	0.22409 (15)	0.8532 (3)	0.6695 (4)	0.0561 (9)	
H5	0.2299	0.8461	0.5882	0.067*	
C9	0.16966 (16)	0.8046 (4)	0.8080 (4)	0.0679 (11)	
H9	0.1381	0.7643	0.8227	0.081*	
C6	0.26283 (16)	0.9246 (4)	0.7689 (4)	0.0698 (11)	
H6	0.2946	0.9649	0.7553	0.084*	
C7	0.25464 (18)	0.9363 (4)	0.8886 (4)	0.0843 (14)	
H7	0.2807	0.9854	0.9566	0.101*	
C8	0.20818 (19)	0.8758 (5)	0.9079 (4)	0.0931 (16)	
H8	0.2027	0.8831	0.9895	0.112*	

### Atomic displacement parameters (Å^2^)

**Table:**

	*U*^11^	*U*^22^	*U*^33^	*U*^12^	*U*^13^	*U*^23^
Cu1	0.0344 (3)	0.0269 (3)	0.0355 (3)	0.000	0.0096 (2)	0.000
O1	0.0352 (12)	0.0329 (10)	0.0490 (13)	−0.0009 (9)	0.0099 (10)	−0.0058 (9)
O2	0.0536 (14)	0.0582 (13)	0.0475 (14)	−0.0047 (11)	0.0216 (12)	−0.0047 (11)
N1	0.0404 (15)	0.0285 (12)	0.0361 (15)	−0.0019 (10)	0.0141 (12)	−0.0016 (10)
C1	0.0395 (18)	0.0278 (14)	0.0384 (18)	0.0010 (13)	0.0061 (14)	0.0090 (13)
C4	0.0333 (17)	0.0362 (15)	0.045 (2)	−0.0030 (13)	0.0084 (15)	−0.0023 (13)
C3	0.0328 (17)	0.0355 (16)	0.051 (2)	−0.0023 (13)	0.0105 (15)	0.0022 (14)
C10	0.0384 (18)	0.0282 (14)	0.0481 (18)	−0.0020 (12)	0.0249 (15)	−0.0033 (12)
C2	0.0355 (17)	0.0387 (16)	0.044 (2)	−0.0061 (13)	0.0098 (15)	0.0046 (13)
C11	0.0487 (19)	0.0355 (16)	0.056 (2)	−0.0084 (14)	0.0275 (17)	−0.0119 (14)
C15	0.067 (3)	0.0255 (15)	0.087 (3)	−0.0056 (15)	0.043 (2)	−0.0087 (14)
C13	0.060 (2)	0.056 (2)	0.040 (2)	−0.0139 (17)	0.0126 (18)	−0.0140 (16)
C14	0.052 (2)	0.0440 (17)	0.039 (2)	−0.0028 (15)	0.0138 (17)	−0.0022 (14)
C12	0.064 (2)	0.0479 (19)	0.061 (2)	−0.0189 (17)	0.030 (2)	−0.0251 (16)
C5	0.054 (2)	0.062 (2)	0.055 (2)	−0.0205 (18)	0.0213 (18)	−0.0090 (17)
C9	0.054 (2)	0.095 (3)	0.061 (3)	−0.031 (2)	0.028 (2)	−0.027 (2)
C6	0.056 (2)	0.075 (2)	0.080 (3)	−0.0317 (19)	0.025 (2)	−0.019 (2)
C7	0.066 (3)	0.108 (3)	0.078 (3)	−0.044 (3)	0.024 (2)	−0.047 (3)
C8	0.080 (3)	0.142 (4)	0.067 (3)	−0.052 (3)	0.039 (3)	−0.049 (3)

### Geometric parameters (Å, º)

**Table:**

Cu1—O1	1.948 (2)	C11—C15	1.433 (4)
Cu1—O1^i^	1.948 (2)	C15—C15^i^	1.342 (7)
Cu1—N1	2.018 (2)	C15—H15	0.9300
Cu1—N1^i^	2.018 (2)	C13—C12	1.363 (5)
O1—C1	1.277 (3)	C13—C14	1.395 (4)
O2—C1	1.232 (3)	C13—H13	0.9300
N1—C14	1.313 (4)	C14—H14	0.9300
N1—C10	1.360 (3)	C12—H12	0.9300
C1—C2	1.479 (4)	C5—C6	1.365 (5)
C4—C9	1.374 (5)	C5—H5	0.9300
C4—C5	1.390 (4)	C9—C8	1.365 (5)
C4—C3	1.466 (4)	C9—H9	0.9300
C3—C2	1.316 (4)	C6—C7	1.368 (5)
C3—H3	0.9300	C6—H6	0.9300
C10—C11	1.404 (4)	C7—C8	1.364 (5)
C10—C10^i^	1.424 (6)	C7—H7	0.9300
C2—H2	0.9300	C8—H8	0.9300
C11—C12	1.395 (5)		
			
N1—Cu1—N1^i^	81.84 (14)	C11—C15—H15	119.1
C1—O1—Cu1	103.84 (18)	C12—C13—C14	119.3 (3)
C14—N1—C10	118.5 (2)	C12—C13—H13	120.4
C14—N1—Cu1	129.00 (19)	C14—C13—H13	120.4
C10—N1—Cu1	112.4 (2)	N1—C14—C13	122.4 (3)
O2—C1—O1	123.2 (3)	N1—C14—H14	118.8
O2—C1—C2	119.8 (3)	C13—C14—H14	118.8
O1—C1—C2	117.0 (3)	C13—C12—C11	120.3 (3)
C9—C4—C5	116.8 (3)	C13—C12—H12	119.8
C9—C4—C3	119.8 (3)	C11—C12—H12	119.8
C5—C4—C3	123.4 (3)	C6—C5—C4	121.9 (3)
C2—C3—C4	128.1 (3)	C6—C5—H5	119.1
C2—C3—H3	115.9	C4—C5—H5	119.1
C4—C3—H3	115.9	C8—C9—C4	121.7 (3)
N1—C10—C11	122.9 (3)	C8—C9—H9	119.1
N1—C10—C10^i^	116.64 (16)	C4—C9—H9	119.1
C11—C10—C10^i^	120.45 (19)	C5—C6—C7	119.6 (3)
C3—C2—C1	123.5 (3)	C5—C6—H6	120.2
C3—C2—H2	118.2	C7—C6—H6	120.2
C1—C2—H2	118.2	C8—C7—C6	119.8 (4)
C12—C11—C10	116.5 (3)	C8—C7—H7	120.1
C12—C11—C15	125.7 (3)	C6—C7—H7	120.1
C10—C11—C15	117.8 (3)	C9—C8—C7	120.3 (4)
C15^i^—C15—C11	121.72 (19)	C9—C8—H8	119.9
C15^i^—C15—H15	119.1	C7—C8—H8	119.9

Symmetry code: (i) −*x*, *y*, −*z*+1/2.

### Hydrogen-bond geometry (Å, º)

**Table:**

*D*—H···*A*	*D*—H	H···*A*	*D*···*A*	*D*—H···*A*
C14—H14···O2^ii^	0.93	2.49	3.176 (5)	131

Symmetry code: (ii) −*x*, −*y*+1, −*z*.
